# Changes in^18^F-FDG uptake measured by PET as a pharmacodynamic end-point in anticancer therapy. How far have we got?

**DOI:** 10.1054/bjoc.2000.1342

**Published:** 2000-07-03

**Authors:** P Price

**Affiliations:** CRC PET Oncology group, MRC Cyclotron Unit, Imperial College School of Medicine, Hammersmith Hospital, London, UK


British Journal of Cancer (2000) 83(3), 281–283
© 2000 Cancer Research Campaign
doi: 10.1054/ bjoc.2000.1342, available online at http://www.idealibrary.com on IDEAL®

Editorial

### Changes in $^{18}$F-FDG uptake measured by PET as a pharmacodynamic end-point in anticancer therapy. How far have we got?

**P Price**

CRC PET Oncology group, MRC Cyclotron Unit, Imperial College School of Medicine, Hammersmith Hospital, London, UK

There has been a lot of hype over the use of PET in oncology, with hopes that it will provide many answers in clinical research. However, now it has been brought to the attention of clinicians and oncology researchers, it is time to get down to the hard work of exploring its potential and defining its true role.

In this edition of the BJC, a paper by Maisey et al (2000) explores the use of $^{18}$F-FDG PET in predicting survival in patients with cancer of the pancreas following systemic chemotherapy. This is one in a series of similar papers that have been published by various groups over the last 4 or 5 years seeking to find the value of FDG PET in measuring response to therapy. So how far have we got?

### What PET is

Positron emission tomography (PET) is a technique which uses radionuclides to label molecules. The molecules can then be imaged in man and this provides quantitative kinetic functional information. The inherent sensitivity and specificity of PET methodology is the most important strength of the technique. Its sensitivity is unrivalled and it can be used to image molecular interactions and pathways, providing quantitative kinetic information down to the sub-pico molar level (Jones, 1996). It lacks the spatial resolution of MRI or CT, but as it is unrivalled in its specificity and kinetic sensitivity, it has a huge future for translation research in oncology (Price, 1997).

Molecular imaging using PET is now being developed to assess in vivo pharmacokinetics and pharmacodynamics in oncology (Young et al, 1999*a*; Saleem et al, 2000). However, currently few groups internationally have the range of expertise in radiochemistry, data analysis, bio-mathematical modelling and kinetic analysis to advance and exploit the technique for these purposes, so progress has been slow. PET methodology is better developed in neurology and psychiatry, but it is still in its infancy in oncology.

### Current diagnostic use of PET

To most of the oncology community, PET is understood to be static $^{18}$F-FDG images of tumours. This is because the vast majority of work that has been performed in PET in oncology has been using $^{18}$F-FDG. FDG (fluorodeoxyglucose) is an analogue of glucose which is taken up into cells by GLUT 1 transporters and trapped, as it is not metabolized by hexokinase. When FDG is labelled with fluorine-18, its kinetics can be imaged with PET and the resultant signal indicates glucose uptake and trapping. If static images are taken at a certain time (say 45 min post-injection) then images show hot-spots in tumours. Much of the work over the last 10 years has looked at the value of FDG as a diagnostic tool. This is on the basis that FDG is preferentially taken up into tumour cells and so from the differential uptake one can define malignant vs non-malignant disease. There are controversies in its use in diagnosis and to what extent it can replace conventional anatomical imaging (Price, 2000).

Cameras for whole-body imaging have been developed in the last few years and so patient scanning can produce a 'soft tissue bone scan'. These images are convincing. However, the technique can produce a number of false-positives, for example, $^{18}$F-FDG is taken up into macrophages, and muscle uptake after exercise can be high. It currently also provides no anatomical resolution. The technique is being promoted by the nuclear medicine community to the oncologists for diagnostic imaging and staging. In the USA there are now five reimbursable indications for $^{18}$F-FDG: investigation of solitary pulmonary nodules, staging of lung cancer, investigation of a rising plasma CEA after colorectal surgery, restaging of melanoma and restaging of lymphoma. The expansion of the use of FDG PET for such diagnosis has been due to FDA approval of $^{18}$F-FDG and its distribution, patient advocacy and arguments of cost-effectiveness. In Europe its use is patchy, being large in Germany, and is dependent on the number of centres in a country and the mechanism of reimbursement. In the UK its use in diagnosis is restricted to a few centres who have equipment and who can devise a funding mechanism to cover the costs. Currently in the UK the cost of a whole-body PET scan is in the region of £800. The argument for the use of $^{18}$F-FDG PET in the UK for diagnosis rests on its priority and cost-effectiveness in a resource-scarce National Health Service.

### PET as a quantitative technique

The field needs to move on to make use of the *quantitative* value of PET. The use of easily achieved static non-objectively quantitative FDG uptake (hot-spots) is insufficient to communicate objective change and is too insensitive to measure the important small changes. Many studies heralding 'hot-spot before, no hot-spot after' in successful treatments such as in chemotherapy for lymphoma do little to demonstrate the unique value of the technique. We need information over and above obvious volume and clinical changes.

The rate of $^{18}$F-FDG uptake into tissues and tumours can be determined and a glucose metabolic rate (MRGlu) measured in μmol min$^{-1}$ ml$^{-1}$ or standard uptake value (SUV) estimated. These quantitative values are being investigated in vivo to see whether FDG PET can be used as a prognostic marker and used to predict survival. As this technique can be repeated before and after treatment it can also be used to look at changes in response to therapy and investigate whether such changes can predict ultimate clinical outcome. As in vivo FDG uptake is broadly related to cell number (Herholz et al, 1993), this can be taken further. Investigations are underway to see whether the change after treatment can be used as a measure of log cell kill and therefore provide a guide to subclinical and clinical response (Price and Jones, 1995).

### PET to measure tumour response: a consensus statement from Europe

The use of FDG PET to measure tumour response is increasing and new papers are being published all the time. In an attempt to be able to pool quantitative data and move the field on more quickly, the EORTC PET Study Group has held a consensus conference in 1999 and reviewed the published and unpublished literature on the subject. They come to recommendations for the use of $^{18}$F-FDG to assess response (Young et al, 1999*b*). In this paper, recommendations are provided on the scanning protocols (such as fasting state of the patients, hydration, medication, etc.), the timing of the scan, the type of scan used and the method of assessment of $^{18}$F-FDG uptake, as well as tumour sampling. The recommendations suggest a definition of $^{18}$F-FDG tumour response in terms of complete, partial metabolic response and stable and progressive metabolic disease. It is only once we have standardized data collection that we can make comparative statements and the field really moves on. These recommendations will now be used for all the EORTC-approved drug development studies using $^{18}$F-FDG PET as a pharmacodynamic measure of anti-proliferative response. The Cancer Research Campaign New Agents Committee are in the process of adopting the EORTC guidelines for its own studies. The National Cancer Institute in the USA will be holding a meeting in June 2000 to decide theirs.

As with all technology that assesses change, the reproducibility of the technique is extremely important. Precious little data on this exists and papers commenting on changes in response to therapy really need control test-re-test data for validity. One PET group in Munich have usefully reported on their reproducibility in 16 patients (Weber et al, 1999). Only by expanding the test-re-test database can we investigate the physiology of the noise in the signal and develop the methodology.

### The potential of $^{18}$F-FDG PET as a metabolic response marker

### *Prediction of clinical response*

From the data so far available in the world, some of it unpublished as yet, it would appear that changes in FDG in response to therapy, when performed accurately, can precede clinical response. This is particularly useful in some instances where these measurements can be taken very early on. In a paper by Brock et al (2000) FDG PET measurements of MRGlu in brain tumours recorded at 7 days were found to be able to predict ultimate clinical and radiological response to chemotherapy recorded at 2 months. There is now published breast cancer data suggesting that this also may be true at other tumour sites (Smith et al 2000, Schelling et al 2000). The use of $^{18}$F-FDG PET in the neoadjuvant setting may be very important – particularly for identifying non-responders early.

### *Measurement of subclinical response*

Whether changes in $^{18}$F-FDG PET can be used as a measure of subclinical response is still unclear. The level with which this technique can measure response that does not appear clinically or radiologically, and yet which is outside the normal reproducibility ranges is unclear. Its use in phase I setting to measure subclinical response has yet to be outlined. An EORTC two-centre study used this technique in a phase I study and is currently in preparation for reporting. One disadvantage may be the range of glucose metabolism in different tumour types which may confound its use in a phase I clinical trial where a range of tumour types are assessed.

### *Measurement of the additive effect of therapy*

Other areas where such a quantitative early readout may be useful is in situations where you are adding therapy. A quantitative readout of the effect of one therapy can then be compared with that of a two-therapy treatment. Currently at our institution we are undertaking a study looking at the additional effects of temozolomide to radiotherapy and assessing whether we can quantitate this additional benefit using $^{18}$F-FDG PET.

### *Measurement of residual disease*

There are many clinical situations where this may prove useful. FDG assessment of 'disease' will not overtake a histological diagnosis, but for instance the identification of residual disease after high-dose radiotherapy may allow small volume boosts in treatment. Work in this area is needed.

### Conclusion

$^{18}$F-FDG PET provides a good signal, can be performed in many centres, and its quantitation is based on mature kinetic modelling. However, there are many difficulties in its use, not least of which is that it is not completely tumour-specific. More work is needed by methodologically competent groups in collaboration with good clinical groups to further define its role. As clinicians, we have a responsibility, as the more we understand of the methodology ourselves the more we will be able to put it to appropriate use and ensure the accuracy and validity of what we are measuring. Advice is available. The CRC drug development section has a pharmacokinetic/pharmacodynamic (PK/PD) committee which provides advice on such end-points. The EORTC have a Functional Imaging group which can also provide information to academia and industry.

### The future

We need more specific markers with high sensitivity which we can use for specific pharmacodynamic end-points. Markers specifically for anti-proliferative therapy, such as $^{11}$C-thymidine and $^{18}$F-FLT, are currently under development. Measures such as $H_2{}^{15}O$ and both $C^{15}O$ to measure tumour perfusion, and blood volume are being developed to assess anti-angiogenic and anti-vascular therapy. Methodological improvements are required, such as high sensitivity cameras, increased ability to co-register images, good radiochemistry and kinetic methodological development.

We also must remember that PET has much wider potential for in vivo pharmacokinetic measurements and the investigation of

 © 2000 Cancer Research Campaign the in vivo mechanisms of action of anti-cancer drugs. We still need more effort put into the methodological development, that leads on to good applications, before this technology will truly reap the rewards of its potential.

### REFERENCES

Brock CS, Young H, O'Reilly SM, Matthews J, Osma S, Evans H, Newlands ES and Price P (2000) Early evaluation of tumour metabolic response using [$^{18}$F]-fluorodeoxyglucose and positron emission tomography: A pilot study following the phase II chemotherapy schedule for temozolomide in recurrent high grade gliomas. *Br J Cancer* **82**(3): 608–615

Herholz K, Pietrzyk U, Voges J, Schroder R, Halber M, Treuer H, Sturn V and Heiss WD (1993) Correlation of glucose consumption and tumour cell density in astrocytomas. A stereotactic PET study. *J Neurosurg* **79**: 853–858

Jones T (1996) The role of positron emission tomography within the spectrum of medical imaging. *Eur J Nucl Med* **23**(2): 211

Maisie et al (2000) Need article title. *Br J Cancer* **82**: 00–00

Price P (1997) Is there a future for PET in oncology? *Eur J Nucl Med* **24**: 587–589

Price P (2000) PET in diagnostic oncology: A necessary tool today. *Eur J Cancer* **36**: 691–693

Price P and Jones T (1995) Can positron emission tomography (PET) be used to detect subclinical response to cancer therapy? *Eur J Cancer* **31A**: 1924–1927

Saleem A, Aboagye EO and Price P (2000) In vivo monitoring of drugs using radiotracer techniques. *Adv Drug Dev Rev* **41**(1): 21–39

Schelling M, Avril N, Näkrig J, Kuhn W, Römer W, Sattler D, Werner M, Dose J, Jännke F, Graett H and Schwaiger M (2000) Positron emission tomography using [$^{18}$F] fluorodeoxyglucose for monitoring primary chemotherapy in breast cancer. *J Clin Oncol* **18**: 1689–1695

Smith IC, Welch A, Hutcheon A, Mitler I, Payne S, Chilcott P, Waiker S, Whitaker T, Ah-See A, Eremin O, Heys S, Gilbert F and Sharp P (2000) Positron emission tomography using [$^{18}$F] fluorodeoxy-P-glucose to predict the pathological response of breast cancer to primary chemotherapy. *J Clin Oncol* **18**: 1676–1688

Weber, Ziegler S, Thodman R, Hanauske AK and Schwaiger M (1999) Reproducibility of metabolic measurements in malignant tumours using FDG PET. *Nucl Med* **35**(13): 1773–1782

Young H, Brock C, Wells and Price (1999*a*) Monitoring response to treatment in the development of anti-cancer drugs using positron emission tomography (PET). *Drug Information Journal* **33**: 237–244

Young H, Baum R and Cremerius U, Herholz K, Hoekstra O, Lammert SMA, Pruim J and Price P (1999*b*) Measurement of clinical and subclinical tumour response using [$^{18}$F]-fluorodeoxyglucose and positron emission tomography: Review and 1999 EORTC recommendations. *Eur J Cancer* **35**(13): 1773–1782

© 2000 Cancer Research Campaign

